# Mitochondria, Microglia, and the Immune System—How Are They Linked in Affective Disorders?

**DOI:** 10.3389/fpsyt.2018.00739

**Published:** 2019-01-09

**Authors:** Carsten Culmsee, Susanne Michels, Stefanie Scheu, Volker Arolt, Udo Dannlowski, Judith Alferink

**Affiliations:** ^1^Institute of Pharmacology and Clinical Pharmacy, University of Marburg, Marburg, Germany; ^2^Center for Mind, Brain and Behavior – CMBB, Marburg, Germany; ^3^Institute of Medical Microbiology and Hospital Hygiene, University of Düsseldorf, Düsseldorf, Germany; ^4^Department of Psychiatry and Psychotherapy, University of Münster, Münster, Germany; ^5^Cells in Motion, Cluster of Excellence, University of Münster, Münster, Germany

**Keywords:** major depressive disorder, immune system, metabolic pathways, mitochondria, immune cells, microglia, neuroinflammation, immunometabolism

## Abstract

Major depressive disorder (MDD) is a severe mood disorder and frequently associated with alterations of the immune system characterized by enhanced levels of circulating pro-inflammatory cytokines and microglia activation in the brain. Increasing evidence suggests that dysfunction of mitochondria may play a key role in the pathogenesis of MDD. Mitochondria are regulators of numerous cellular functions including energy metabolism, maintenance of redox and calcium homeostasis, and cell death and therefore modulate many facets of the innate immune response. In depression-like behavior of rodents, mitochondrial perturbation and release of mitochondrial components have been shown to boost cytokine production and neuroinflammation. On the other hand, pro-inflammatory cytokines may influence mitochondrial functions such as oxidative phosphorylation, production of adenosine triphosphate, and reactive oxygen species, thereby aggravating inflammation. There is strong interest in a better understanding of immunometabolic pathways in MDD that may serve as diagnostic markers and therapeutic targets. Here, we review the interaction between mitochondrial metabolism and innate immunity in the pathophysiology of MDD. We specifically focus on immunometabolic processes that govern microglial and peripheral myeloid cell functions, both cellular components involved in neuroinflammation in depression-like behavior. We finally discuss microglial polarization and associated metabolic states in depression-associated behavior and in MDD.

## Introduction

Major depression is a serious mood disorder and characterized by marked functional impairment and increased health care utilization (1, 2). In particular, major depressive disorder (MDD) is estimated to affect more than 300 million people worldwide and treatment resistant depression occurs in about 20–30% of patients (3). Therefore, a better understanding of the underlying mechanisms is warranted to improve the therapeutic options in MDD.

Although the pathophysiology of MDD is not yet fully understood, genetic and environmental factors have been identified as major risk factors for the development of depression (4–6). In addition, a plethora of findings point toward an association between inflammation and depression. Immune alterations such as increased levels of circulating pro-inflammatory cytokines and polymorphisms in immune–associated genes have been frequently found in depressed individuals. Additional observations that immunotherapy with type I interferons may induce depressive symptoms and that depression-like “sickness behavior” in rodents is caused by treatment with inflammatory mediators underscored the bidirectional relationship between the immune response and depression (7–11). Accordingly, the “inflammation hypothesis of depression” has been proposed over 20 years ago (12, 13). The multi-faceted inflammatory process in depression has been reviewed in detail before (13–18). In this review, we focus on the intricate interplay between metabolic processes and innate immunity in the pathophysiology of MDD. We further discuss the link between mitochondrial dysfunction and neuroinflammation in depression-associated behavior in the rodent model. Finally, we highlight the concept that specific metabolic processes are associated with distinct microglial activation states that may contribute to the pathogenesis of depression.

## Innate Immune Response in Depression

### Mitochondrial Function in the Innate Immune Response

**Figure 1:**
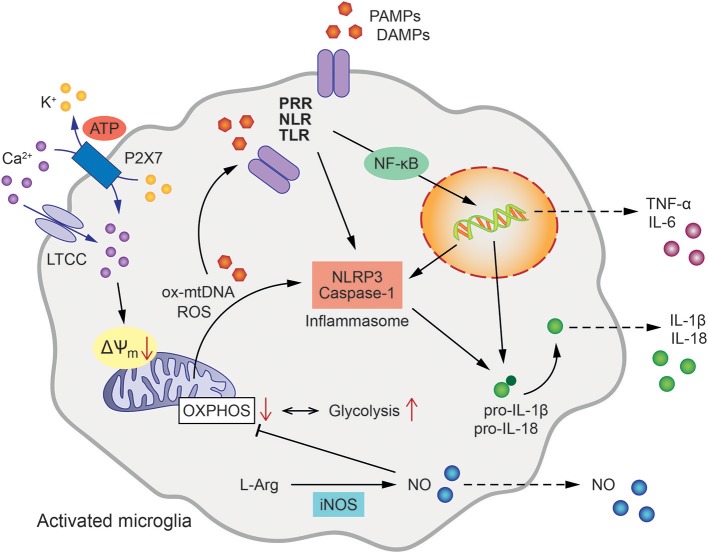
Mitochondrial involvement in microglial activation and inflammatory signaling. Microglial activation pathways and inflammatory cytokine release are initiated by the binding of pathogen- or damage-associated molecular patterns (PAMPs/DAMPs) to intracellular or membrane-bound pattern recognition receptors (PRR), such as NOD-like receptors (NLR) or Toll-like receptors (TLR). This triggers NLRP3 and Caspase-1 assembly to form the inflammasome leading to the processing of pro-IL-1β as well as pro-IL-18 to active IL-1β and IL-18, respectively. In addition, oxidized mitochondrial DNA (ox-mtDNA) and mitochondrial reactive oxygen species (ROS) are canonical activators of inflammasome formation. Tumor necrosis factor (TNF) and Interleukin-6 (IL-6) are generated via nuclear factor NF-κB-dependent transcriptional activation. The oxidation of L-arginine (L-Arg) by inducible nitric oxide synthase (iNOS) produces NO (Nitric oxide), which in turn inhibits oxidative phosphorylation (OXPHOS) shifting the energy metabolism of the cell toward glycolysis. Ca^2+^ influx via L-type calcium channels (LTCC) and ATP-binding purinoceptors (P2X7) results in the loss of mitochondrial membrane potential (ΔΨm), enhanced mitochondrial ROS formation and further contributes to inflammasome formation and pro-inflammatory activation of microglia.

The innate immune system represents the first line of defense against invading microbial pathogens and comprises a variety of cell types, molecules, and signaling cascades (19). Myeloid cells are the cellular components of innate immunity and represent a heterogeneous group of bone marrow (BM)—derived cells including monocytes/macrophages, dendritic cells (DCs), and granulocytes (20). Within minutes of encountering pathogens, highly conserved pathogen-associated molecular patterns (PAMPs) bind to pattern recognition receptors (PRR) expressed within the cytosol or on membranes of innate immune cells, such as RIG-I-like receptors (RLR), NOD-like receptors (NLR), or Toll-like receptors (TLR) (21) (Figure 1). Activated PRR can trigger the release of cytokines, chemokines, and additional inflammatory factors via various intracellular pathways to ultimately control infection (22). Even in the absence of overt pathogenic infection, cell damage or stress responses may alert the innate immune response and induce a “sterile inflammatory immune response.” In this context, endogenous or “self-molecules” (e.g., high mobility group box 1, S100 proteins, RNA, and DNA) are recognized as danger signals when released into the extracellular space. These damage-associated molecular pattern molecules (DAMPs) trigger innate immune responses also via binding of PRRs (23).

Multiple lines of evidence strongly suggest that mitochondrial integrity and function, and innate immunity are closely interlinked processes. Mitochondria are intracellular organelles required for numerous cellular functions including energy metabolism, regulation of reactive oxygen species (ROS) signaling, Ca^2+^ homeostasis, and apoptosis. In addition, several mitochondrial components such as adenosine triphosphate (ATP), N-formyl peptides or mitochondrial DNA (mtDNA) function as DAMPs and are sensed by distinct PRRs thereby promoting an inflammatory response (24). Accordingly, it has been demonstrated in humans that injury induces release of N-formyl peptides and mtDNA into the circulation and activates neutrophils via binding of formyl peptide receptor-1 and TLR9, respectively (25). Studies in mice demonstrated that mtDNA aggravated the inflammatory response, while inflammation was reduced in animals deficient for TLR9 or the adaptor protein, myeloid differentiation primary response gene 88 (Myd88) (26). Furthermore, also ATP has been found to induce mitochondrial dysfunction, enhanced generation of ROS, and apoptosis, resulting in cytosolic release of oxidized mtDNA, that binds to and activates the NLR family pyrin domain containing 3 (NLRP3) inflammasome (27).

The inflammasome is a multi-protein signaling complex that triggers caspase-1-dependent secretion of the pro-inflammatory cytokines interleukin-1β (IL-1β) and IL-18 (25, 28, 29). A particular role has been described for intracellular mtDNA and mitochondria-derived ROS in pathways that activate the inflammasome (30–32). For example, mtDNA accumulation in the cytosol of macrophages was identified as a prerequisite for caspase 1-dependent IL-1β release in response to combined lipopolysaccharide (LPS) and ATP exposure. The essential role of mitochondria in this process was further demonstrated by depletion of autophagic proteins that enhanced the accumulation of dysfunctional mitochondria in macrophages thereby increasing mitochondrial ROS production and susceptibility to stimulation by LPS and ATP (30, 33). Further, extracellular ATP can induce NLRP3 inflammasome activation through engagement of P2x7 receptors and downstream mitochondrial dysfunction. The purinergic P2x7 receptor is an ATP-gated ion channel that is expressed by virtually all immune cell subsets and its activation has been associated with inflammation (34). A recent study demonstrated in macrophages that K^+^ efflux and Ca^2+^ influx through P2x7 were required for sustained reduction of the mitochondrial membrane potential and generation of mitochondrial ROS formation upstream of NLRP3 inflammasome assembly and pyroptotic cell death (35). Earlier studies pointed toward ROS as the key regulators of the NLRP inflammasome in response to PAMPS and DAMPS, such as oxidized mtDNA or other DAMPs resulting from metabolic dysregulation (36, 37). More recent studies, however, underscored the importance of new mtDNA synthesis for NLRP3 inflammasome activation. According to these findings, LPS-induced TLR signaling via MyD88 and Toll/interleukin-1 receptor domain-containing adaptor protein inducing interferon beta (TRIF) triggers transcription of the mitochondrial cytidine/uridine monophosphate kinase-2 (CMPK2). CMPK2 belongs to a family of nucleotide kinases that are required for mtDNA synthesis and production of oxidized mtDNA fragments that ultimately act as activating ligands for the NLRP3 inflammasome complex in stimulated macrophages (38).

### Innate Immune Responses in MDD

Dysregulation of innate immune responses has been linked to stress-associated psychiatric disorders such as MDD (16, 39–43). A plethora of studies and meta-analyses have demonstrated that patients with MDD frequently show elevated levels of TNF, IL-6, as well as the T helper cell differentiation cytokine IL-12 (44–48). Stress may induce the activation of the innate immune system and stressful experiences such as adverse childhood events induce long-term alterations of the immune response and increase the susceptibility to depression (49–55). In analogy to depression, exposure to early life stressors in humans has been shown to elevate blood levels of pro-inflammatory cytokines (56). Mechanistically, pro-inflammatory cytokines can activate the hypothalamic–pituitary–adrenal axis leading to hypercortisolism and increased glucocorticoid receptor resistance, both mechanisms involved in the etiology of MDD (57). In addition, pro-inflammatory cytokines modulate the tryptophan–kynurenine pathway and enhance synthesis of the neurotoxic N-methyl-D-aspartate (NMDA) glutamate receptor agonist quinolinic acid and 3-hydroxykynurenine with detrimental effects on brain function (41). Recent studies support the idea that inflammation contributes to depression in a subgroup of patients characterized by enhanced disease severity and potentially neurovegetative symptoms. In addition, somatic comorbidities associated with an ongoing inflammatory process and elevated circulating inflammatory factors have shown better treatment responses to anti-inflammatory agents in MDD [for review see Raison and Miller (42)].

It is important to note, however, that altered blood cytokine levels as discussed here are not specific for affective disorders, but have also been found elevated in post-traumatic stress disorders (PTSD) (58, 59), obsessive-compulsive disorders (OCD) (60) or eating disorders (61). Additionally, low grade inflammation and cytokine elevation play a role in a number of physical diseases for example metabolic diseases such as diabetes and obesity as hallmarks of the metabolic syndrome. It has also to be considered that overall altered cytokine levels may result from distinct immune activation patterns on the level of immune cell subsets. Therefore, in addition to the analysis of overall cytokine levels in the peripheral blood, characterization of cytokine and receptor expression profiles of specific immune cell subsets (i.e., immune signatures) may better represent an individual‘s psychiatric disease risk and progression. Longitudinal studies are required in MDD patients and healthy participants, including those with familial genetic risk and exposure to early life stress (e.g., childhood maltreatment) to identify such immune signatures. In the multicenter cohort study FOR2107, we established a large-scale multi-parameter flow cytometry screen for characterization of immune activation profiles on a single cell level with prognostic potential in MDD patients with genetic (G), environmental (E), or GxE risk factors. In this cohort study, established immune signatures in patients are now compared to those identified in peripheral immune cells and microglia of the CNS in genetic and behavioral rat models of depression in defined GxE risk settings (62). This translational approach will provide a better understanding of the functional impact of (neuro-) inflammatory responses in MDD and the mechanisms by which GxE alters immune activation profiles and the risk to develop MDD.

### Microglia in MDD and Depression-Associated Behavior

In analogy to humans, also in rodent models, depression-associated behavior after stress exposure is frequently associated with “low-grade immune activation” characterized by enhanced levels of circulating pro-inflammatory cytokines and immigration of myeloid immune cells into the brain (63–66). Specifically, trafficking of “inflammatory” Ly6C^hi^ monocytes that co-express CC chemokine receptor 2 (CCR2), the receptor for the CC chemokine ligand 2 (CCL2), to the brain has been shown to promote neuroinflammation in the stress response (67–69). The treatment of mice with the TLR4 ligand LPS is well-known to induce an innate immune response and trigger sickness behavior, i.e., anhedonia and weight loss. Mice deficient for the inflammatory caspase-1 exhibit enhanced resistance to LPS-induced depressive-like behavior underscoring the involvement of the inflammasome in depression (70). Also pretreatment with an NLRP3 inflammasome inhibitor abrogated the depressive-like behaviors induced by LPS in mice (71). Interesting findings further identified TRIF, one of the key mediators of oxidized mtDNA production in NLRP3 inflammasome activation, as an important inflammatory signaling mediator of LPS-induced sickness behavior through regulation of CCL2 in the hypothalamus (72). This CCR2-CCL2 signaling mechanism may thus link metabolic and behavioral adaptation to inflammation in the brain (73).

Peripheral immune alterations are closely linked to microglia activation that plays a prominent role in the pathogenesis of MDD and depression-associated behavior (65, 74–77). Microglia express PRRs and thus recognize PAMPs and DAMPs. Upon ligand binding to PRRs, microglia acquire an amoeboid-like phenotype, migrate to inflammatory sites, and release pro-inflammatory cytokines (e.g., IL-1β, IL-6, IL-18, TNF), chemokines, and neurotoxic factors such as nitric oxide (NO) generated by the inducible NO synthase (iNOS) and ROS (78–81). Classically activated M1 microglia are induced by stimulation with LPS, granulocyte-macrophage colony-stimulating factor (GM-CSF) or interferon-γ (IFN-γ) and express enhanced levels of major histocompatibility complex (MHC) class II, and CD86. They are involved in the defense against pathogens but may also occur in stress responses. Upon alternative activation with IL-1 and anti-inflammatory IL-10, M2 microglia express arginase-1 (Arg-1), the chitinase-like protein Ym1, Fizz1 (found in inflammatory zone), anti-inflammatory cytokines, extracellular matrix proteins, and glucocorticoids (82). In analogy to peripheral macrophages, the M2 microglia phenotype has further been sub-classified into M2a, M2b, and M2c activation states, each subtype specifically equipped to contribute to immune regulation, phagocytosis, and/or tissue repair [for review, see Singhal and Baune (79)].

To study microglial activation in depression *in vivo*, positron emission tomography (PET) imaging studies using various PET ligands for the microglial marker translocator protein 18 kDa (TSPO) have been conducted. TSPO predominantly localizes to the outer mitochondrial membrane and is expressed in brain microglia (83). Depression-associated elevations in TSPO in the prefrontal cortex, insula, and anterior cingulate cortex have been correlated with the severity and duration of depression (84). Post-mortem and PET imaging studies further identified microglia activation in individuals who committed suicide (74, 85–87). However, also negative results demonstrating lack of microglia activation or even suppressed microglia activation states have been reported in depressed individuals [for review, see Yirmiya et al. (88)].

Several studies investigated substances that may affect M1 to M2 polarization in microglia. For example, inhibition of the JAK/STAT pathway is known to suppress M1-associated downstream genes in inflammatory CNS disorders (89). Treatment with the PPARγ agonist, pioglitazone, has been shown to cause a phenotypic switch of M1 microglia to the anti-inflammatory M2 state in various CNS disease models and to mediate antidepressant properties in several studies (90). Furthermore, Glatiramer acetate, approved for the treatment of relapsing-remitting MS, mediates neuroprotective effects by inducing an anti-inflammatory microglial M2 phenotype and thus harbors potential for treatment of MDD (91). A study in cultured mouse microglial cells demonstrated that the antidepressant selective serotonin reuptake inhibitors (SSRI) fluoxetine and S-citalopram can inhibit M1 activation and enhanced M2 activation of these immune cells *in vitro* (92). With regard to microglia activation in depression-associated behavior, several studies have been conducted in rodent models. One study demonstrated that mice exposed to social defeat stress, an established stress/depression model, exhibit microglia activation and increased expression of microglial-derived pro-inflammatory cytokines specifically in brain regions associated with fear and anxiety (31). Furthermore, inhibition of microglial activation or NLRP3 deletion has been proven to impair stress-induced alterations associated with depression in rodents (93). Treatment with substances mediating antidepressant effects has further been shown to suppress classical microglial activation and increased the microglial M2 markers in the brain of C57BL/6 mice exposed to chronic mild stress (94) Moreover, anti-inflammatory effects of tricyclic antidepressants, SSRI, and lithium have been described *in vivo*, in animal models of IFNα-induced sickness behavior and inflammation-induced cytokine production in the brain (45, 95).

As has been discussed in this chapter, exposure to early life stressors represents a risk factor for MDD and depression-like behavior and is associated with alterations of the innate immune response. Elevated blood levels of pro-inflammatory cytokines in depressed individuals may affect microglia activation, a pathophysiological hallmark of major depression.

## Involvement of Mitochondria in the Neurobiology of Affective Disorders

### Mitochondrial Impairments Associated With Depression

Intracellular and intercellular mechanisms of stress adaptation in the brain such as in the course of MDD lead to a significant increase in energy demand (96). In neural cells mitochondria are pivotal for energy production through oxidative phosphorylation that converts the chemical energy stored in glucose to ATP. Furthermore, mitochondria are essential for Ca^2+^ homeostasis, generation of ROS, neuronal outgrowth and differentiation, synaptic plasticity, and cell death signaling. Thus, they are highly important for cellular resilience and stress adaptation in the brain. More recent reports suggested a role for mitochondrial dysfunction and related major hallmarks of cellular stress, such as impaired redox balance and deregulation of intracellular Ca^2+^ homeostasis in the development of MDD and bipolar disorders (BD) (97–100).

While affective disorders such as MDD or BD are not considered as classic mitochondrial diseases, emerging evidence suggests a substantial link between mitochondrial dysfunction and these disorders in genetic and behavioral animal models, as well as in patients (99, 101, 102). For example, patients suffering from mitochondrial diseases caused by genetic alterations affecting mitochondrial metabolism frequently develop symptoms of MDD, BD, psychosis, and personality changes (103–105). Further, mood disorders are often prevalent years before the onset of cognitive and motor symptoms in patients later diagnosed with neurodegenerative diseases, such as Alzheimer's, Parkinson's, and Huntington's disease (106, 107), which all feature mitochondrial dysfunction in neurons as a major hallmark of the underlying pathology (107–109). Concurring reports are derived from genetic studies as well as post-mortem brain analysis, brain imaging or biomarker studies in patients diagnosed with affective disorders, and in the respective animal models (99, 110). Mitochondrial impairments are characterized by morphological, biochemical, and functional hallmarks which all contribute to disturbed energy metabolism, but also to reduced Ca^2+^ buffering, loss of membrane potential, and increased mitochondrial ROS production. Finally, fatal mitochondrial dysfunction can result in disruption of the mitochondrial membrane and release of pro-apoptotic proteins such as cytochrome c or apoptosis-inducing factor (AIF) which mediate caspase-dependent or caspase-independent cell death, respectively.

Disturbed oxidative phosphorylation (OXPHOS) and reduced mitochondrial ATP production may significantly contribute to impaired neuronal plasticity and neurogenesis which are considered hallmarks in the neurobiology of depression (102). Several studies detected lower ATP levels in the brain tissue of MDD patients compared to healthy controls (111, 112). Similar correlations of depressive behavior and mitochondrial dysfunction in energy supply were confirmed in animal models of depression. In a mouse model of chronic restraint stress depressive behavior in the tail suspension and forced swim tests was associated with decreased oxygen consumption rate in isolated brain mitochondria (113). Further, impaired mitochondrial respiration and additional features of mitochondrial damage such as altered mitochondrial membrane potential and changes in the mitochondrial ultrastructure were also detected in other rodent models of depression induced by chronic mild stress such as learned helplessness in mice (114) or anhedonia in rats (115). Interestingly, treatment with the antidepressant fluoxetine reversed the depressive behavior and restored ATP production in brain tissue in a rat model of unpredictive chronic stress (116).

Mitochondria are highly dynamic organelles that undergo permanent fission and fusion processes allowing for the transport, reorganization, and regeneration of these organelles within the cells. In a model of streptozotocin-induced diabetes in mice, the associated depressive behavior was accompanied by increased expression of mitochondrial fission genes fission protein 1 (Fis1) and dynamin-related protein 1 (Drp1), and a decreased expression of mitochondrial fusion genes mitofusin 1 (Mfn1), mitofusin 2 (Mfn2), and optical atrophy 1 (Opa1) in the brain tissue (117). Further, the DISC1 protein is an important regulator of mitochondrial dynamics and mediates the transport, fusion, and regeneration of these organelles in neuronal axons and dendrites (118). Pathological DISC1 isoforms disrupt mitochondrial dynamics leading to abnormal neuronal development and DISC1 mutations have been implicated in major mental disorders including MDD and BD (119). Intact mitochondrial fission and fusion dynamics are also important for the proper cristae formation, respiratory functions of these organelles, and quality control through mitophagy. Impairments in the structural dynamics lead to reduced energy supply, accumulation of dysfunctional mitochondria and increased ROS production. These phenomena are closely associated with both, enhanced inflammatory responses (120, 121) and the risk of psychiatric disorders, including MDD (122).

In particular, oxidative stress has been frequently linked to the pathophysiology of depression. In MDD patients and in animal models, decreased levels of antioxidants and antioxidant enzymes were detected, suggesting an impaired antioxidant defense associated with depressive behavior. For example, in a rat model of restraint stress glutathione levels were significantly decreased in the brain tissue for weeks after stress exposure (123). In neurons, glutathione depletion leads to increased lipid peroxidation and the activation of pro-apoptotic signaling pathways that involve the activation and mitochondrial translocation of BH3-interacting domain death agonist (BID) and the fission-inducing GTPase Drp1. Upon mitochondrial transactivation, these proteins mediate mitochondrial fission, mitochondrial ROS production, ATP depletion, and disruption of the mitochondrial membrane (124–126). Notably, lipid peroxidation was enhanced in mouse brain tissue after restraint stress (123), and increased oxidative damage and altered expression levels of the electron transport chain complex I were also detected in brain tissue of MDD patients (127). Inhibition of complex I leads to a rapid increase in mitochondrial ROS formation which further impairs mitochondrial respiration, integrity, and function. As outlined before, complex I inhibition and the associated increase in mitochondrial ROS formation and oxidized mtDNA have been established as a trigger mechanism for inflammatory responses in macrophages through activation of the NLRP3 (36–38).

### The Risk Gene CACNA1C and Mitochondrial Dysfunction

How mitochondrial functions are affected by genetic risk factors and environmental stress in the context of affective disorders is an emerging field of research. The trigger mechanisms of mitochondrial pathology, such as oxidative stress, impaired intracellular Ca^2+^ homeostasis, and molecular signaling pathways causing loss of mitochondrial function and integrity have been associated with the pathology of affective disorders. In particular, recent findings closely connected the psychiatric risk gene CACNA1C to mitochondrial dysfunction in conditions of oxidative stress. CACNA1C codes for the α1c subunit of the L-type Ca^2+^ channel (LTCC) Cav1.2, and has been identified by several genome-wide association studies as one of the strongest and most replicable risk factors for MDD and BD (128). In cultured mouse neuronal cells, reduction of CACNA1C expression or pharmacological inhibition of LTCC prevented excessive ROS formation, mitochondrial damage and ATP depletion, and rescued the neurons from cell death in a model of oxidative stress (129, 130). Our data corroborate earlier reports demonstrating that CACNA1C depletion or pharmacological LTCC inhibition was associated with antidepressant-like behavior and resilience to chronic stress, while activation of CACNA1C was detrimental for synaptic plasticity and cognitive functions (131, 132). In gene/genetic x environmental risk interactions, mitochondrial dysfunction may represent a converging point of the complex interdependent processes of energy metabolism, cellular stress, and calcium homeostasis in the neurobiology of affective disorders.

Overall, increased cellular ROS levels and the ensuing oxidative stress may be cause as well as consequence of mitochondrial dysfunction and metabolic impairments involved in neuroinflammatory responses in the neurobiology of depression. The according signaling pathways may serve as future therapeutic targets. Similar to therapeutic effects on innate immune responses and mitochondrial impairments, antidepressants attenuate parameters of oxidative stress in MDD patients and animal models [for review, see Allen et al. (102) and Adzic et al. (133)]. Further, a recent study exposed functional perturbations of apoptotic mitochondrial stress signaling induced by BID as a potential therapeutic target in rodent models of depression (134). Targeting such mechanisms of mitochondrial damage may provide novel therapeutic approaches in both, age-related disorders of the nervous system and psychiatric disorders (135–137). Systematic studies investigating the impact of genetic risk factors and environmental stress on mitochondrial functions and morphological alterations are highly warranted for a better understanding of the proposed link between the course of psychiatric disorders and mitochondrial demise.

## Immunometabolism and Neuroinflammatory Responses in MDD

### Metabolic Programs in M1 and M2 Like Macrophages and Microglia

The research field termed immunometabolism has significantly advanced our understanding on the link between immunological and metabolic processes in immune cell differentiation and effector function. Naïve as well as activated immune cells require the capacity to produce ATP as energy supply for cellular function and it has been demonstrated that myeloid cells primarily use glycolysis as a source of ATP that represents a major mechanism of pro-inflammatory adaptation (138, 139). It is well-established that inflammatory factors such as pro-inflammatory cytokines influence mitochondrial function and can shift ATP production from OXPHOS to glycolysis. In this regard, TNF produced e.g., by activated microglia inhibited OXPHOS and concomitantly induced enhanced mitochondrial ROS production (120).

Immunometabolism may fine-tune myeloid cell functions and thereby influence activation states and polarization of myeloid cells. In accordance, M1 and M2 macrophages have been linked to distinct metabolic programs (139). It has been shown that classically activated M1 macrophages exhibit enhanced aerobic glycolysis and increased pentose phosphate pathway (PPP), while mitochondrial fatty acid oxidation (FAO), the Krebs-cycle, and OXPHOS were reduced (140, 141). This metabolic shift in M1 cells allows for conserving and generating metabolites necessary for pro-inflammatory activation, cell proliferation and concomitant supply of the required amount of ATP. For example, succinate from the inactive Krebs-cycle activates hypoxia-inducible factor 1-alpha (HIF1-α) which stimulates IL-1β production, and, together with the increased glycolysis, supports cell activity and survival in hypoxic-inflammatory environments (141). Enhanced NO production through oxidation of L-arginine by iNOs activity is another hallmark of activated M1 macrophages and microglia (142, 143). NO reduces Krebs-cycle activity through inhibition of the pyruvate dehydrogenase, i.e., by reducing the production of acetyl-CoA from pyruvate (144, 145). Further, increased NO levels can also reversibly inhibit OXPHOS through inhibition of the mitochondrial cytochrome oxidase.

Similar to peripheral immune cells, LPS stimulation of mouse microglial cell lines and primary microglia revealed a metabolic switch from mitochondrial respiration to glycolysis (146) (and own observations in primary rat microglia). The LPS-mediated activation of microglia was accompanied by increased lactate production and activation of glycolysis-driving enzymes such as hexokinase, glucose-6-phosphate dehydrogenase, phosphofructokinase-1, and lactate dehydrogenase. The metabolic shift upon TLR activation in macrophages and microglia appears to occur in two steps that allows for utilizing OXPHOS, glycolysis, and the PPP simultaneously in the first phase, while glycolytic metabolism and the PPP support survival and pro-inflammatory activity after full M1 transformation (146). In contrast, anti-inflammatory M2 macrophages, supporting e.g., wound-healing, utilize fatty acid oxidation as the primary energy source which results in generation of acetyl-CoA that is shuttled to the catabolic Krebs-cycle in the mitochondrial matrix (144, 147). This metabolic state represents the phenotype of resident macrophages and features reduced glucose utilization and the synthesis of ornithine and polyamines to promote cell proliferation and tissue repair, collagen synthesis, fibrosis, and tissue remodeling (142). In cultured mouse microglia, induction of the M2 phenotype by exposure to IL-4 was also accompanied by reduced glucose consumption and lactate production, and mitochondrial respiration was preserved to control levels in non-stimulated cells (146). These findings are in contrast to peripheral human macrophages, where IL-4 stimulation enhanced glucose uptake, fatty acid metabolism, and mitochondrial biogenesis (148), thus pointing to differences in the M2 states between these two immune cell populations. More insight into the molecular mechanisms of microglia polarization is required to identify potential targets for pharmacological intervention at the level of mitochondrial metabolism or at the level of cytokine regulation and signaling (149).

### Metabolic Programs in PMBCs

Up to now there is limited knowledge on the effect of mitochondria-derived metabolic pathways on (neuro-)inflammation in MDD. Furthermore, the impact of pro-inflammatory cytokines on mitochondrial functioning in depression is yet unresolved. During neuroinflammation in depression-associated behavior, inflammatory mediators such as TNF produced by activated microglia and brain-infiltrating immune cells trigger intracellular signaling cascades that can alter mitochondrial metabolism, ROS formation, and programmed cell death as outlined before. In contrast to microglia, which are hardly accessible from MDD patients, peripheral blood mononuclear cells (PBMCs) may provide an accessible source of the mitochondrial pool with relevance to alterations of mitochondrial functions in the brain. It has been shown recently in non-human primates that the mitochondrial bioenergetics profile of blood monocytes and platelets is positively related to frontal cortex mitochondrial function and metabolism (150). Brain mitochondrial dysfunction, in turn, is significantly involved in the pathophysiology of psychiatric disorders as supported by a growing body of literature (102, 151). In fact, a few studies already assessed mitochondrial function in circulating blood cells of psychiatric patients (152–154). For example, basal and maximal mitochondrial respiration was significantly lower in platelets (153) as well as in PBMCs (152) of depressed patients vs. healthy controls. Fresh intact platelets of depressive patients in partial remission showed decreased basal and maximal respiration, whereas the ratio of both values remained unchanged compared to healthy individuals (153). Basal and maximal mitochondrial respiration, and ATP production were significantly lower in cryopreserved PBMCs of female patients with a current diagnosis of major depression (152). As outlined before, compromised mitochondrial metabolism often leads to excess superoxide production thereby modulating redox-sensitive inflammatory pathways and inducing oxidative stress, which most likely play a role in MDD pathophysiology (155, 156). The Bioenergetic Health Index (BHI) comprises several parameters of a person's respiration profile and overall mitochondrial function (157, 158). By considering the spare respiratory capacity, the BHI may even have predictive value for the development of affective disorders because it may already identify alterations in mitochondrial performance before cellular energy failure occurs.

In this chapter we reviewed studies providing compelling evidence for metabolic re-programming in peripheral innate immune cells and in microglia upon activation. This is characterized by a switch from mitochondrial respiration to glycolysis and the PPP in the pro-inflammatory “M1” phenotype and, in contrast, to enhanced utilization of fatty acid and acetyl-CoA shuttling to the Krebs-cycle in anti-inflammatory M2 macrophages/microglia. The pro-inflammatory M1 phenotype has been associated with enhanced disease status in MDD, whereas a switch toward M2-activated microglia was associated with the therapeutic effect of antidepressants. Whether bioenergetic profiles of peripheral immune cells could serve as predictive biomarkers in affective disorders or even as therapeutic target with relevance for both, peripheral immune cells and microglia in the brain, requires further investigation.

## Summary

As discussed in this review, certain metabolic pathways may determine microglia differentiation to shape the effector function of these cells. Consequently, manipulating these pathways may constitute a novel target to combat detrimental inflammatory responses in affective disorders. For example, the potential to promote an M1 to M2 shift in microglia during neuroinflammation in MDD may have beneficial therapeutic implications. In patients, PBMCs may be a valuable surrogate model of brain function and established mitochondrial perturbations in PBMCs may serve as biomarkers for neuropsychiatric disorders. In most studies, impaired mitochondrial respiration in the PBMCs was linked to an enhanced risk for or already established psychiatric disorders in the donor patients. Limitations in the overall comparability of the reported findings are attributed to differences in study cohort characteristics, antidepressant medication, cell type, cell storage, and detection methods of mitochondrial function. Whether mitochondrial dysfunction precedes the onset of psychiatric disorders has not been investigated in detail so far. Therefore, it remains to be elucidated, if changes in mitochondrial bioenergetics are already present in healthy individuals with psychiatric disease-relevant genetic or environmental risk factors and thus can serve as prognostic marker before clinical symptoms manifest. However, the impact of metabolic regulation in immune cell activation on the pathophysiology of depression and the question how increasing knowledge on immunometabolism could be translated into potential therapies for affective disorders remains to be answered.

## Author Contributions

All authors listed have made a substantial, direct and intellectual contribution to the work, and approved it for publication.

### Conflict of Interest Statement

VA is member of advisory boards and/or gave presentations for the following companies: Astra-Zeneca, Allergan, Janssen-Organon, Lundbeck, Otsuka, Servier, and Trommsdorff. JA gave a presentation for the company Servier. The remaining authors declare that the research was conducted in the absence of any commercial or financial relationships that could be construed as a potential conflict of interest

## Abbreviations

AIF: Apoptosis inducing factor
Arg-1: Arginase-1
ATP: Adenosine triphosphate
BD: Bipolar disorder
BHI: Bioenergetic health index
BID: BH3-interacting domain death agonist
BM: Bone marrow
CCL2: Chemokine ligand 2
CCR2: Chemokine receptor 2
CMPK2: Cytidine/uridine monophosphate kinase-2
DAMP: Damage-associated molecular pattern
DC: Dendritic cell
ΔΨm: Mitochondrial membrane potential
DISC1: Disrupted in schizophrenia 1
Drp1: Dynamin-related protein 1
FAO: Fatty acid oxidation
GM-CSF: Granulocyte-macrophage colony-stimulating factor
GxE: Gene-environment interaction
HIF1-α: Hypoxia-inducible factor 1-alpha
iNOS: Inducible nitric oxide synthase
L-Arg: L-Arginine
LPS: Lipopolysaccharide
LTCC: L-type calcium channel
MDD: Major depressive disorder
Mfn: Mitofusin
mtDNA: Mitochondrial deoxyribonucleic acid
Myd88: Myeloid differentiation primary response gene 88
NF: Nuclear factor
NLR: NOD-like receptor
NLRP3: NLR family pyrin domain containing 3
NMDA: N-Methyl-D-aspartate
NO: Nitric oxide
OCD: Obsessive-compulsive disorder
OPA1: Optical atrophy 1
OXPHOS: Oxidative phosphorylation
P2X7: ATP-binding purinoceptor
PAMP: Pathogen-associated molecular pattern
PBMCs: Peripheral blood mononuclear cells
PET: Positron emission tomography
PPP: Pentose phosphate pathway
PRR: Pattern recognition receptor
PTSD: Post-traumatic stress disorder
RLR: RIG-I-like receptor
ROS: Reactive oxygen species
SSRI: Selective serotonin reuptake inhibitors
TLR: Toll-like receptor
TRIF: Toll/interleukin-1 receptor domain-containing adaptor protein inducing interferon beta
TSPO: Microglial translocator protein.

## References

[1] Greden JF. The burden of recurrent depression: causes, consequences, and future prospects. J Clin Psychiatry (2001) 62(Suppl. 22):5–9. 11599650

[2] Greenberg PE Kessler RC Birnbaum HG Leong SA Lowe SW Berglund PA. The economic burden of depression in the United States: how did it change between 1990 and 2000? J Clin Psychiatry (2003) 64:1465–75. 10.4088/JCP.v64n1211 14728109

[3] Nemeroff CB. Prevalence and management of treatment-resistant depression. J Clin Psychiatry (2007) 68(Suppl. 8):17–25. 17640154

[4] Kupfer DJ Frank E Phillips ML. Major depressive disorder: new clinical, neurobiological, and treatment perspectives. Lancet (2012) 379:1045–55. 10.1016/S0140-6736(11)60602-8 22189047 PMC3397431

[5] Direk N Williams S Smith JA Ripke S Air T Amare AT. An analysis of two genome-wide association meta-analyses identifies a new locus for broad depression phenotype. Biol Psychiatry (2017) 82:322–9. 10.1016/j.biopsych.2016.11.013 28049566 PMC5462867

[6] Yu C Baune BT Wong ML Licinio J. Investigation of short tandem repeats in major depression using whole-genome sequencing data. J Affect Disord. (2018) 232:305–9. 10.1016/j.jad.2018.02.046 29501989

[7] Mills NT Scott JG Wray NR Cohen-Woods S Baune BT. Research review: the role of cytokines in depression in adolescents: a systematic review. J Child Psychol Psychiatry Allied Discip. (2013) 54:816–35. 10.1111/jcpp.12080 24027786

[8] Prager G Hadamitzky M Engler A Doenlen R Wirth T Pacheco-Lopez G Amygdaloid signature of peripheral immune activation by bacterial lipopolysaccharide or staphylococcal enterotoxin B. J Neuroimmune Pharmacol. (2013) 8:42–50. 10.1007/s11481-012-9373-0 22639228

[9] Raison CL Capuron L Miller AH. Cytokines sing the blues: inflammation and the pathogenesis of depression. Trends Immunol. (2006) 27:24–31. 10.1016/j.it.2005.11.006 16316783 PMC3392963

[10] Capuron L Miller AH. Cytokines and psychopathology: lessons from interferon-alpha. Biol Psychiatry (2004) 56:819–24. 10.1016/j.biopsych.2004.02.009 15576057

[11] Dantzer R. Cytokine, sickness behavior, and depression. Neurol Clin. (2006) 24:441–60. 10.1016/j.ncl.2006.03.003 16877117 PMC2909644

[12] Maes M. Evidence for an immune response in major depression: a review and hypothesis. Prog Neuro Psychopharmacol Biol Psychiatry (1995) 19:11–38. 10.1016/0278-5846(94)00101-M 7708925

[13] Maes M. The cytokine hypothesis of depression: inflammation, oxidative & nitrosative stress (IO&NS) and leaky gut as new targets for adjunctive treatments in depression. Neuro Endocrinol Lett. (2008) 29:287–91. 18580840

[14] Leonard BE. Inflammation and depression: a causal or coincidental link to the pathophysiology? Acta Neuropsychiatr. (2018) 30:1–16. 10.1017/neu.2016.69 28112061

[15] Bauer ME Teixeira AL. Inflammation in psychiatric disorders: what comes first? Ann NY Acad Sci. (2018). [Epub ahead of print]. 10.1111/nyas.13712 29752710

[16] Eyre HA Stuart MJ Baune BT. A phase-specific neuroimmune model of clinical depression. Prog Neuro Psychopharmacol Biol Psychiatry (2014) 54:265–74. 10.1016/j.pnpbp.2014.06.011 24999185

[17] Miller AH Raison CL. Are anti-inflammatory therapies viable treatments for psychiatric disorders?: Where the rubber meets the road. JAMA Psychiatry (2015) 72:527–8. 10.1001/jamapsychiatry.2015.22 25853989 PMC5542670

[18] Bufalino C Hepgul N Aguglia E Pariante CM. The role of immune genes in the association between depression and inflammation: a review of recent clinical studies. Brain Behav Immun. (2013) 31:31–47. 10.1016/j.bbi.2012.04.009 22580182

[19] Medzhitov R Shevach EM Trinchieri G Mellor AL Munn DH Gordon S. Highlights of 10 years of immunology in Nature Reviews Immunology. Nat Rev Immunol. (2011) 11:693–702. 10.1038/nri3063 21941295 PMC3703536

[20] Geissmann F Manz MG Jung S Sieweke MH Merad M Ley K. Development of monocytes, macrophages, and dendritic cells. Science (2010) 327:656–61. 10.1126/science.1178331 20133564 PMC2887389

[21] Takeuchi O Akira S. Pattern recognition receptors and inflammation. Cell (2010) 140:805–20. 10.1016/j.cell.2010.01.022 20303872

[22] Iwasaki A Medzhitov R. Control of adaptive immunity by the innate immune system. Nat. Immunol. (2015) 16:343–53. 10.1038/ni.3123 25789684 PMC4507498

[23] Rock KL Latz E Ontiveros F Kono H. The sterile inflammatory response. Ann Rev Immunol. (2010) 28:321–42. 10.1146/annurev-immunol-030409-101311 20307211 PMC4315152

[24] Krysko DV Agostinis P Krysko O Garg AD Bachert C Lambrecht BN. Emerging role of damage-associated molecular patterns derived from mitochondria in inflammation. Trends Immunol. (2011) 32:157–64. 10.1016/j.it.2011.01.005 21334975

[25] Zhang Q Raoof M Chen Y Sumi Y Sursal T Junger W. Circulating mitochondrial DAMPs cause inflammatory responses to injury. Nature (2010) 464:104–7. 10.1038/nature08780 20203610 PMC2843437

[26] Boyapati RK Tamborska A Dorward DA Ho GT. Advances in the understanding of mitochondrial DNA as a pathogenic factor in inflammatory diseases. F1000Res. (2017) 6:169. 10.12688/f1000research.10397.1 28299196 PMC5321122

[27] Shimada K Crother TR Karlin J Dagvadorj J Chiba N Chen S. Oxidized mitochondrial DNA activates the NLRP3 inflammasome during apoptosis. Immunity (2012) 36:401–14. 10.1016/j.immuni.2012.01.009 22342844 PMC3312986

[28] Sutterwala FS Ogura Y Szczepanik M Lara-Tejero M Lichtenberger GS Grant EP. Critical role for NALP3/CIAS1/Cryopyrin in innate and adaptive immunity through its regulation of caspase-1. Immunity (2006) 24:317–27. 10.1016/j.immuni.2006.02.004 16546100

[29] Heneka MT McManus RM Latz E. Inflammasome signalling in brain function and neurodegenerative disease. Nat Rev Neurosci. (2018) 19:610–21. 10.1038/s41583-018-0055-7 30206330

[30] Nakahira K Haspel JA Rathinam VA Lee SJ Dolinay T Lam HC. Autophagy proteins regulate innate immune responses by inhibiting the release of mitochondrial DNA mediated by the NALP3 inflammasome. Nat Immunol. (2011) 12:222–30. 10.1038/ni.1980 21151103 PMC3079381

[31] Zhou R Yazdi AS Menu P Tschopp J. A role for mitochondria in NLRP3 inflammasome activation. Nature (2011) 469:221–5. 10.1038/nature09663 21124315

[32] Fleshner M Frank M Maier SF. Danger signals and inflammasomes: stress-evoked sterile inflammation in mood disorders. Neuropsychopharmacology (2017) 42:36–45. 10.1038/npp.2016.125 27412959 PMC5143484

[33] Sadatomi D Nakashioya K Mamiya S Honda S Kameyama Y Yamamura Y. Mitochondrial function is required for extracellular ATP-induced NLRP3 inflammasome activation. J Biochem. (2017) 161:503–12. 10.1093/jb/mvw098 28096454

[34] Deussing JM Arzt E. P2X7 receptor: a potential therapeutic target for depression? Trends Mol Med. (2018) 24:736–47. 10.1016/j.molmed.2018.07.005 30093269

[35] Yaron JR Gangaraju S Rao MY Kong X Zhang L Su F. K(+) regulates Ca(2+) to drive inflammasome signaling: dynamic visualization of ion flux in live cells. Cell Death Dis. (2015) 6:e1954. 10.1038/cddis.2015.277 26512962 PMC5399176

[36] Tschopp J Schroder K. NLRP3 inflammasome activation: the convergence of multiple signalling pathways on ROS production? Nat Rev Immunol. (2010) 10:210–5. 10.1038/nri2725 20168318

[37] Schroder K Tschopp J. The inflammasomes. Cell (2010) 140:821–32. 10.1016/j.cell.2010.01.040 20303873

[38] Zhong Z Liang S Sanchez-Lopez E He F Shalapour S Lin XJ. New mitochondrial DNA synthesis enables NLRP3 inflammasome activation. Nature (2018) 560:198–203. 10.1038/s41586-018-0372-z 30046112 PMC6329306

[39] Gibney SM Drexhage HA. Evidence for a dysregulated immune system in the etiology of psychiatric disorders. J Neuroimmune Pharmacol. (2013) 8:900–20. 10.1007/s11481-013-9462-8 23645137

[40] Maes M Yirmyia R Noraberg J Brene S Hibbeln J Perini G The inflammatory & neurodegenerative (I&ND) hypothesis of depression: leads for future research and new drug developments in depression. Metabol Brain Dis. (2009) 24:27–53. 10.1007/s11011-008-9118-1 19085093

[41] Dantzer R O'Connor JC Lawson MA Kelley KW. Inflammation-associated depression: from serotonin to kynurenine. Psychoneuroendocrinology (2011) 36:426–36. 10.1016/j.psyneuen.2010.09.012 21041030 PMC3053088

[42] Raison CL Miller AH. Is depression an inflammatory disorder? Curr Psychiatry Rep. (2011) 13:467–75. 10.1007/s11920-011-0232-0 21927805 PMC3285451

[43] Grosse L Carvalho LA Wijkhuijs AJ Bellingrath S Ruland T Ambree O. Clinical characteristics of inflammation-associated depression: monocyte gene expression is age-related in major depressive disorder. Brain Behav Immun. (2015) 44:48–56. 10.1016/j.bbi.2014.08.004 25150007

[44] Dowlati Y Herrmann N Swardfager W Liu H Sham L Reim EK. A meta-analysis of cytokines in major depression. Biol. Psychiatry (2010) 67:446–57. 10.1016/j.biopsych.2009.09.033 20015486

[45] Kohler CA Freitas TH Stubbs B Maes M Solmi M Veronese N. Peripheral alterations in cytokine and chemokine levels after antidepressant drug treatment for major depressive disorder: systematic review and meta-analysis. Mol Neurobiol. (2018) 55:4195–206. 10.1007/s12035-017-0632-1 28612257

[46] Baune BT Konrad C Grotegerd D Suslow T Birosova E Ohrmann P. Interleukin-6 gene (IL-6): a possible role in brain morphology in the healthy adult brain. J Neuroinflamm. (2012) 9:125. 10.1186/1742-2094-9-125 22695063 PMC3464888

[47] Baune BT Konrad C Grotegerd D Suslow T Ohrmann P Bauer J. Tumor necrosis factor gene variation predicts hippocampus volume in healthy individuals. Biol. Psychiatry (2012) 72:655–62. 10.1016/j.biopsych.2012.04.002 22554453

[48] Goldsmith DR Rapaport MH Miller BJ. A meta-analysis of blood cytokine network alterations in psychiatric patients: comparisons between schizophrenia, bipolar disorder and depression. Mol Psychiatry (2016) 21:1696–709. 10.1038/mp.2016.3 26903267 PMC6056174

[49] Steptoe A Hamer M Chida Y. The effects of acute psychological stress on circulating inflammatory factors in humans: a review and meta-analysis. Brain Behav Immun. (2007) 21:901–12. 10.1016/j.bbi.2007.03.011 17475444

[50] Redlich R Stacey D Opel N Grotegerd D Dohm K Kugel H. Evidence of an IFN-gamma by early life stress interaction in the regulation of amygdala reactivity to emotional stimuli. Psychoneuroendocrinology (2015) 62:66–73. 10.1016/j.psyneuen.2015.08.008 26313134

[51] Opel N Redlich R Zwanzger P Grotegerd D Arolt V Heindel W. Hippocampal atrophy in major depression: a function of childhood maltreatment rather than diagnosis? Neuropsychopharmacology (2014) 39:2723–31. 10.1038/npp.2014.145 24924799 PMC4200502

[52] Hazel NA Hammen C Brennan PA Najman J. Early childhood adversity and adolescent depression: the mediating role of continued stress. Psychol Med. (2008) 38:581–9. 10.1017/S0033291708002857 18261247

[53] Krishnan V Nestler EJ. The molecular neurobiology of depression. Nature (2008) 455:894–902. 10.1038/nature07455 18923511 PMC2721780

[54] Otte C Gold SM Penninx BW Pariante CM Etkin A Fava M et al. Major depressive disorder. Nat Rev Dis Primers (2016) 2:16065. 10.1038/nrdp.2016.65 27629598

[55] Schedlowski M Engler H Grigoleit JS. Endotoxin-induced experimental systemic inflammation in humans: a model to disentangle immune-to-brain communication. Brain Behav Immun. (2014) 35:1–8. 10.1016/j.bbi.2013.09.015 24491305

[56] Cattaneo A Macchi F Plazzotta G Veronica B Bocchio-Chiavetto L Riva MA. Inflammation and neuronal plasticity: a link between childhood trauma and depression pathogenesis. Front Cell Neurosci. (2015) 9:40. 10.3389/fncel.2015.00040 25873859 PMC4379909

[57] Cohen S Janicki-Deverts D Doyle WJ Miller GE Frank E Rabin BS. Chronic stress, glucocorticoid receptor resistance, inflammation, and disease risk. Proc Natl Acad Sci USA. (2012) 109:5995–9. 10.1073/pnas.1118355109 22474371 PMC3341031

[58] Waheed A Dalton B Wesemann U Ibrahim MAA Himmerich H. A systematic review of interleukin-1beta in post-traumatic stress disorder: evidence from human and animal studies. J Interferon Cytokine Res. (2018) 38:1–11. 10.1089/jir.2017.0088 29328883

[59] Hussein S Dalton B Willmund GD Ibrahim MAA Himmerich H. A systematic review of tumor necrosis factor-alpha in post-traumatic stress disorder: evidence from human and animal studies. Psychiatr Danub. (2017) 29:407–20. 10.24869/psyd.2017.407 29197197

[60] Rao NP Venkatasubramanian G Ravi V Kalmady S Cherian A Yc JR. Plasma cytokine abnormalities in drug-naive, comorbidity-free obsessive-compulsive disorder. Psychiatry Res. (2015) 229:949–52. 10.1016/j.psychres.2015.07.009 26187339

[61] Dalton B Bartholdy S Robinson L Solmi M Ibrahim MAA Breen G. A meta-analysis of cytokine concentrations in eating disorders. J Psychiatr Res. (2018) 103:252–64. 10.1016/j.jpsychires.2018.06.002 29906710

[62] Kircher T Wohr M Nenadic I Schwarting R Schratt G Alferink J. Neurobiology of the major psychoses: a translational perspective on brain structure and function-the FOR2107 consortium. Eur Arch Psychiatry Clin Neurosci. (2018). [Epub ahead of print]. 10.1007/s00406-018-0943-x 30267149

[63] Krishnan V Berton O Nestler E. The use of animal models in psychiatric research and treatment. Am J Psychiatry (2008) 165:1109. 10.1176/appi.ajp.2008.08071076 18765492

[64] Toyoda A. Social defeat models in animal science: what we have learned from rodent models. Anim Sci J. (2017) 88:944–52. 10.1111/asj.12809 28436163 PMC5518448

[65] Ramirez K Fornaguera-Trias J Sheridan JF. Stress-induced microglia activation and monocyte trafficking to the brain underlie the development of anxiety and depression. Curr Top Behav Neurosci. (2017) 31:155–72. 10.1007/7854_2016_25 27352390

[66] Engler H Bailey MT Engler A Sheridan JF. Effects of repeated social stress on leukocyte distribution in bone marrow, peripheral blood and spleen. J Neuroimmunol. (2004) 148:106–15. 10.1016/j.jneuroim.2003.11.011 14975591

[67] Wohleb ES Powell ND Godbout JP Sheridan JF. Stress-induced recruitment of bone marrow-derived monocytes to the brain promotes anxiety-like behavior. J Neurosci. (2013) 33:13820–33. 10.1523/JNEUROSCI.1671-13.2013 23966702 PMC3755721

[68] Zheng X Ma S Kang A Wu M Wang L Wang Q. Chemical dampening of Ly6C(hi) monocytes in the periphery produces anti-depressant effects in mice. Sci Rep. (2016) 6:19406. 10.1038/srep19406 26783261 PMC4725984

[69] Ambree O Ruland C Scheu S Arolt V Alferink J. Alterations of the innate immune system in susceptibility and resilience after social defeat stress. Front Behav Neurosci. (2018) 12:141. 10.3389/fnbeh.2018.00141 30057531 PMC6053497

[70] Li MX Zheng HL Luo Y He JG Wang W Han J. Gene deficiency and pharmacological inhibition of caspase-1 confers resilience to chronic social defeat stress via regulating the stability of surface AMPARs. Mol Psychiatry (2018) 23:556–68. 10.1038/mp.2017.76 28416811 PMC5822452

[71] Zhang Y Liu L Peng YL Liu YZ Wu TY Shen XL. Involvement of inflammasome activation in lipopolysaccharide-induced mice depressive-like behaviors. CNS Neurosci Ther. (2014) 20:119–24. 10.1111/cns.12170 24279434 PMC6493120

[72] Burfeind KG Zhu X Levasseur PR Michaelis KA Norgard MA Marks DL. TRIF is a key inflammatory mediator of acute sickness behavior and cancer cachexia. Brain Behav Immun. (2018) 73:364–74. 10.1016/j.bbi.2018.05.021 29852290 PMC6129432

[73] Le Thuc O Cansell C Bourourou M Denis RG Stobbe K Devaux N. Central CCL2 signaling onto MCH neurons mediates metabolic and behavioral adaptation to inflammation. EMBO Rep. (2016) 17:1738–52. 10.15252/embr.201541499 27733491 PMC5283585

[74] Reus GZ Fries GR Stertz L Badawy M Passos IC Barichello T. The role of inflammation and microglial activation in the pathophysiology of psychiatric disorders. Neuroscience (2015) 300:141–54. 10.1016/j.neuroscience.2015.05.018 25981208

[75] Tay TL Bechade C D'Andrea I St-Pierre MK Henry MS Roumier A. Microglia gone rogue: impacts on psychiatric disorders across the lifespan. Front Mol Neurosci. (2017) 10:421. 10.3389/fnmol.2017.00421 29354029 PMC5758507

[76] Kalkman HO Feuerbach D. Antidepressant therapies inhibit inflammation and microglial M1-polarization. Pharmacol Ther. (2016) 163:82–93. 10.1016/j.pharmthera.2016.04.001 27101921

[77] Reader BF Jarrett BL McKim DB Wohleb ES Godbout JP Sheridan JF. Peripheral and central effects of repeated social defeat stress: monocyte trafficking, microglial activation, and anxiety. Neuroscience (2015) 289:429–42. 10.1016/j.neuroscience.2015.01.001 25596319 PMC4536813

[78] Ransohoff RM Brown MA. Innate immunity in the central nervous system. J Clin Invest. (2012) 122:1164–71. 10.1172/JCI58644 22466658 PMC3314450

[79] Singhal G Baune BT. Microglia: an interface between the loss of neuroplasticity and depression. Front Cell Neurosci. (2017) 11:270. 10.3389/fncel.2017.00270 28943841 PMC5596091

[80] Tay TL Savage JC Hui CW Bisht K Tremblay ME. Microglia across the lifespan: from origin to function in brain development, plasticity and cognition. J Physiol. (2017) 595:1929–45. 10.1113/JP272134 27104646 PMC5350449

[81] Napoli I Neumann H. Microglial clearance function in health and disease. Neuroscience (2009) 158:1030–8. 10.1016/j.neuroscience.2008.06.046 18644426

[82] Amici SA Dong J Guerau-de-Arellano M. Molecular mechanisms modulating the phenotype of macrophages and microglia. Front Immunol. (2017) 8:1520. 10.3389/fimmu.2017.01520 29176977 PMC5686097

[83] Yrondi A Aouizerate B El-Hage W Moliere F Thalamas C Delcourt N. Assessment of translocator protein density, as marker of neuroinflammation, in major depressive disorder: a pilot, multicenter, comparative, controlled, Brain PET Study (INFLADEP Study). Front. Psychiatry (2018) 9:326. 10.3389/fpsyt.2018.00326 30087626 PMC6066663

[84] Setiawan E Attwells S Wilson AA Mizrahi R Rusjan PM Miler L. Association of translocator protein total distribution volume with duration of untreated major depressive disorder: a cross-sectional study. Lancet Psychiatry (2018) 5:339–47. 10.1016/S2215-0366(18)30048-8 29496589

[85] Norden DM Godbout JP. Review: microglia of the aged brain: primed to be activated and resistant to regulation. Neuropathol Appl Neurobiol. (2013) 39:19–34. 10.1111/j.1365-2990.2012.01306.x 23039106 PMC3553257

[86] Steiner J Bielau H Brisch R Danos P Ullrich O Mawrin C. Immunological aspects in the neurobiology of suicide: elevated microglial density in schizophrenia and depression is associated with suicide. J Psychiatr Res. (2008) 42:151–7. 10.1016/j.jpsychires.2006.10.013 17174336

[87] Schnieder TP Trencevska I Rosoklija G Stankov A Mann JJ Smiley J. Microglia of prefrontal white matter in suicide. J Neuropathol Exp Neurol. (2014) 73:880–90. 10.1097/NEN.0000000000000107 25101704 PMC4141011

[88] Yirmiya R Rimmerman N Reshef R. Depression as a microglial disease. Trends Neurosci. (2015) 38:637–58. 10.1016/j.tins.2015.08.001 26442697

[89] Liu Y Holdbrooks AT De Sarno P Rowse AL Yanagisawa LL McFarland BC. Therapeutic efficacy of suppressing the Jak/STAT pathway in multiple models of experimental autoimmune encephalomyelitis. J Immunol. (2014) 192:59–72. 10.4049/jimmunol.1301513 24323580 PMC3934829

[90] Colle R de Larminat D Rotenberg S Hozer F Hardy P Verstuyft C. PPAR-gamma agonists for the treatment of major depression: a review. Pharmacopsychiatry (2017) 50:49–55. 10.1055/s-0042-120120 27978584

[91] English C Aloi JJ. New FDA-approved disease-modifying therapies for multiple sclerosis. Clin Ther. (2015) 37:691–715. 10.1016/j.clinthera.2015.03.001 25846320

[92] Su F Yi H Xu L Zhang Z. Fluoxetine and S-citalopram inhibit M1 activation and promote M2 activation of microglia *in vitro*. Neuroscience (2015) 294:60–8. 10.1016/j.neuroscience.2015.02.028 25711936

[93] Alcocer-Gomez E Ulecia-Moron C Marin-Aguilar F Rybkina T Casas-Barquero N Ruiz-Cabello J. Stress-induced depressive behaviors require a functional NLRP3 inflammasome. Mol Neurobiol. (2016) 53:4874–82. 10.1007/s12035-015-9408-7 26362308

[94] Zhao Q Wu X Yan S Xie X Fan Y Zhang J. The antidepressant-like effects of pioglitazone in a chronic mild stress mouse model are associated with PPARgamma-mediated alteration of microglial activation phenotypes. J Neuroinflam. (2016) 13:259. 10.1186/s12974-016-0728-y 27716270 PMC5051050

[95] Kenis G Maes M. Effects of antidepressants on the production of cytokines. Int J Neuropsychopharmacol. (2002) 5:401–12. 10.1017/S1461145702003164 12466038

[96] Herculano-Houzel S. Scaling of brain metabolism with a fixed energy budget per neuron: implications for neuronal activity, plasticity and evolution. PLoS ONE (2011) 6:e17514. 10.1371/journal.pone.0017514 21390261 PMC3046985

[97] Anglin RE Garside SL Tarnopolsky MA Mazurek MF Rosebush PI. The psychiatric manifestations of mitochondrial disorders: a case and review of the literature. J Clin Psychiatry (2012) 73:506–12. 10.4088/JCP.11r07237 22579150

[98] Kato T. Role of mitochondrial DNA in calcium signaling abnormality in bipolar disorder. Cell Calcium (2008) 44:92–102. 10.1016/j.ceca.2007.11.005 18177933

[99] Manji H Kato T Di Prospero NA Ness S Beal MF Krams M. Impaired mitochondrial function in psychiatric disorders. Nat Rev Neurosci. (2012) 13:293–307. 10.1038/nrn3229 22510887

[100] Moylan S Eyre HA Maes M Baune BT Jacka FN Berk M. Exercising the worry away: how inflammation, oxidative and nitrogen stress mediates the beneficial effect of physical activity on anxiety disorder symptoms and behaviours. Neurosci Biobehav Rev. (2013) 37:573–84. 10.1016/j.neubiorev.2013.02.003 23415701

[101] Morava E Kozicz T. Mitochondria and the economy of stress (mal)adaptation. Neurosci Biobehav Rev. (2013) 37:668–80. 10.1016/j.neubiorev.2013.02.005 23415702

[102] Allen J Romay-Tallon R Brymer KJ Caruncho HJ Kalynchuk LE. Mitochondria and mood: mitochondrial dysfunction as a key player in the manifestation of depression. Front Neurosci. (2018) 12:386. 10.3389/fnins.2018.00386 29928190 PMC5997778

[103] Fattal O Link J Quinn K Cohen BH Franco K. Psychiatric comorbidity in 36 adults with mitochondrial cytopathies. CNS Spectr. (2007) 12:429–38. 10.1017/S1092852900015303 17545953

[104] Cataldo AM McPhie DL Lange NT Punzell S Elmiligy S Ye NZ. Abnormalities in mitochondrial structure in cells from patients with bipolar disorder. Am J Pathol. (2010) 177:575–85. 10.2353/ajpath.2010.081068 20566748 PMC2913344

[105] Petschner P Gonda X Baksa D Eszlari N Trivaks M Juhasz G. Genes linking mitochondrial function, cognitive impairment and depression are associated with endophenotypes serving precision medicine. Neuroscience (2018) 370:207–17. 10.1016/j.neuroscience.2017.09.049 28987512

[106] Woolley JD Khan BK Murthy NK Miller BL Rankin KP. The diagnostic challenge of psychiatric symptoms in neurodegenerative disease: rates of and risk factors for prior psychiatric diagnosis in patients with early neurodegenerative disease. J Clin Psychiatry (2011) 72:126–33. 10.4088/JCP.10m06382oli 21382304 PMC3076589

[107] Galindo MF Ikuta I Zhu X Casadesus G Jordan J. Mitochondrial biology in Alzheimer's disease pathogenesis. J Neurochem. (2010) 114:933–45. 10.1111/j.1471-4159.2010.06814.x 20492350

[108] Henchcliffe C Beal MF. Mitochondrial biology and oxidative stress in Parkinson disease pathogenesis. Nat Clin Pract Neurol. (2008) 4:600–9. 10.1038/ncpneuro0924 18978800

[109] Turner C Schapira AH. Mitochondrial matters of the brain: the role in Huntington's disease. J Bioenerg Biomembr. (2010) 42:193–8. 10.1007/s10863-010-9290-y 20480217

[110] Scaini G Santos PM Benedet J Rochi N Gomes LM Borges LS. Evaluation of Krebs cycle enzymes in the brain of rats after chronic administration of antidepressants. Brain Res Bull. (2010) 82:224–7. 10.1016/j.brainresbull.2010.03.006 20347017

[111] Moretti A Gorini A Villa RF. Affective disorders, antidepressant drugs and brain metabolism. Mol Psychiatry (2003) 8:773–85. 10.1038/sj.mp.4001353 12931205

[112] Martins-de-Souza D Guest PC Harris LW Vanattou-Saifoudine N Webster MJ Rahmoune H. Identification of proteomic signatures associated with depression and psychotic depression in post-mortem brains from major depression patients. Transl Psychiatry (2012) 2:e87. 10.1038/tp.2012.13 22832852 PMC3309534

[113] Kambe Y Miyata A. Potential involvement of the mitochondrial unfolded protein response in depressive-like symptoms in mice. Neurosci Lett. (2015) 588:166–71. 10.1016/j.neulet.2015.01.006 25576703

[114] Gong Y Chai Y Ding JH Sun XL Hu G. Chronic mild stress damages mitochondrial ultrastructure and function in mouse brain. Neurosci Lett. (2011) 488:76–80. 10.1016/j.neulet.2010.11.006 21070835

[115] Gamaro GD Streck EL Matte C Prediger ME Wyse AT Dalmaz C. Reduction of hippocampal Na+, K+-ATPase activity in rats subjected to an experimental model of depression. Neurochem Res. (2003) 28:1339–44. 10.1023/A:1024988113978 12938855

[116] Wen L Jin Y Li L Sun S Cheng S Zhang S. Exercise prevents raphe nucleus mitochondrial overactivity in a rat depression model. Physiol Behav. (2014) 132:57–65. 10.1016/j.physbeh.2014.04.050 24813829

[117] Chen C Wang Y Zhang J Ma L Gu J Ho G. Contribution of neural cell death to depressive phenotypes of streptozotocin-induced diabetic mice. Dis Models Mech. (2014) 7:723–30. 10.1242/dmm.016162 24764190 PMC4036479

[118] Norkett R Modi S Birsa N Atkin TA Ivankovic D Pathania M. DISC1-dependent regulation of mitochondrial dynamics controls the morphogenesis of complex neuronal dendrites. J Biol Chem. (2016) 291:613–29. 10.1074/jbc.M115.699447 26553875 PMC4705382

[119] Kato T. Molecular genetics of bipolar disorder and depression. Psychiatry Clin Neurosci. (2007) 61:3–19. 10.1111/j.1440-1819.2007.01604.x 17239033

[120] Urrutia PJ Mena NP Nunez MT. The interplay between iron accumulation, mitochondrial dysfunction, and inflammation during the execution step of neurodegenerative disorders. Front Pharmacol. (2014) 5:38. 10.3389/fphar.2014.00038 24653700 PMC3948003

[121] Gardner A Boles RG. Beyond the serotonin hypothesis: mitochondria, inflammation and neurodegeneration in major depression and affective spectrum disorders. Prog Neuro Psychopharmacol Biol Psychiatry (2011) 35:730–43. 10.1016/j.pnpbp.2010.07.030 20691744

[122] Cordeiro JL Marques W Hallak JE Osorio FL. Charcot-Marie-Tooth disease, psychiatric indicators and quality of life: a systematic review. ASN Neuro (2014) 6:185–92. 10.1042/AN20130048 24654889 PMC4034707

[123] Madrigal JL Olivenza R Moro MA Lizasoain I Lorenzo P Rodrigo J. Glutathione depletion, lipid peroxidation and mitochondrial dysfunction are induced by chronic stress in rat brain. Neuropsychopharmacology (2001) 24:420–9. 10.1016/S0893-133X(00)00208-6 11182537

[124] Neitemeier S Jelinek A Laino V Hoffmann L Eisenbach I Eying R. BID links ferroptosis to mitochondrial cell death pathways. Redox Biol. (2017) 12:558–70. 10.1016/j.redox.2017.03.007 28384611 PMC5382034

[125] Jelinek A Heyder L Daude M Plessner M Krippner S Grosse R. Mitochondrial rescue prevents glutathione peroxidase-dependent ferroptosis. Free Radic Biol Med. (2018) 117:45–57. 10.1016/j.freeradbiomed.2018.01.019 29378335

[126] Grohm J Kim SW Mamrak U Tobaben S Cassidy-Stone A Nunnari J. Inhibition of Drp1 provides neuroprotection *in vitro* and *in vivo*. Cell Death Diff. (2012) 19:1446–58. 10.1038/cdd.2012.18 22388349 PMC3422469

[127] Ben-Shachar D Karry R. Neuroanatomical pattern of mitochondrial complex I pathology varies between schizophrenia, bipolar disorder and major depression. PLoS ONE (2008) 3:e3676. 10.1371/journal.pone.0003676 18989376 PMC2579333

[128] Lee SH Ripke S Neale BM Faraone SV Purcell SM Perlis RH. Genetic relationship between five psychiatric disorders estimated from genome-wide SNPs. Nat Genet. (2013) 45:984–94. 10.1038/ng.2711 23933821 PMC3800159

[129] Michels S Wohr M Schwarting RK Culmsee C. Psychiatric risk gene Cacna1c determines mitochondrial resilience against oxidative stress in neurons. Cell Death Dis. (2018) 9:645. 10.1038/s41419-018-0676-9 29844355 PMC5974319

[130] Michels S Ganjam GK Martins H Schratt GM Wohr M Schwarting RKW. Downregulation of the psychiatric susceptibility gene Cacna1c promotes mitochondrial resilience to oxidative stress in neuronal cells. Cell Death Discov. (2018) 4:54. 10.1038/s41420-018-0061-6 29760952 PMC5945680

[131] Kabir ZD Lee AS Burgdorf CE Fischer DK Rajadhyaksha AM Mok E. Cacna1c in the prefrontal cortex regulates depression-related behaviors via REDD1. Neuropsychopharmacology (2017) 42:2032–42. 10.1038/npp.2016.271 27922594 PMC5561335

[132] Yoshimizu T Pan JQ Mungenast AE Madison JM Su S Ketterman J Functional implications of a psychiatric risk variant within CACNA1C in induced human neurons. Mol Psychiatry (2015) 20:284 10.1038/mp.2014.181 25623946

[133] Adzic M Brkic Z Bulajic S Mitic M Radojcic MB. Antidepressant action on mitochondrial dysfunction in psychiatric disorders. Drug Dev Res. (2016) 77:400–6. 10.1002/ddr.21332 27539538

[134] Malkesman O Austin DR Tragon T Henter ID Reed JC Pellecchia M. Targeting the BH3-interacting domain death agonist to develop mechanistically unique antidepressants. Mol Psychiatry (2012) 17:770–80. 10.1038/mp.2011.77 21727899 PMC3274661

[135] Cardoso AL Costa P de Almeida LP Simoes S Plesnila N Culmsee C. Tf-lipoplex-mediated c-Jun silencing improves neuronal survival following excitotoxic damage *in vivo*. J Control. Release (2010) 142:392–403. 10.1016/j.jconrel.2009.11.004 19913061

[136] Culmsee C Plesnila N. Targeting Bid to prevent programmed cell death in neurons. Biochem Soc Trans. (2006) 34 (Pt 6):1334–40. 10.1042/BST0341334 17073814

[137] Pereira C Chavarria V Vian J Ashton MM Berk M Marx W. Mitochondrial agents for bipolar disorder. Int J Neuropsychopharmacol. (2018) 21:550–69. 10.1093/ijnp/pyy018 29596661 PMC6007750

[138] Kelly B O'Neill LA. Metabolic reprogramming in macrophages and dendritic cells in innate immunity. Cell Res. (2015) 25:771–84. 10.1038/cr.2015.68 26045163 PMC4493277

[139] Zhu L Zhao Q Yang T Ding W Zhao Y. Cellular metabolism and macrophage functional polarization. Int Rev Immunol. (2015) 34:82–100. 10.3109/08830185.2014.969421 25340307

[140] Haschemi A Kosma P Gille L Evans CR Burant CF Starkl P. The sedoheptulose kinase CARKL directs macrophage polarization through control of glucose metabolism. Cell Metab. (2012) 15:813–26. 10.1016/j.cmet.2012.04.023 22682222 PMC3370649

[141] Galvan-Pena S O'Neill LA. Metabolic reprograming in macrophage polarization. Front Immunol. (2014) 5:420. 10.3389/fimmu.2014.00420 25228902 PMC4151090

[142] Mills CD. M1 and M2 macrophages: oracles of health and disease. Crit Rev Immunol. (2012) 32:463–88. 10.1615/CritRevImmunol.v32.i6.10 23428224

[143] Rath M Muller I Kropf P Closs EI Munder M. Metabolism via arginase or nitric oxide synthase: two competing arginine pathways in macrophages. Front Immunol. (2014) 5:532. 10.3389/fimmu.2014.00532 25386178 PMC4209874

[144] De Santa F Vitiello L Torcinaro A Ferraro E. The role of metabolic remodeling in macrophage polarization and its effect on skeletal muscle regeneration. Antioxid Redox Signal. (2018). [Epub ahead of print]. 10.1089/ars.2017.742 30070144

[145] Klimaszewska-Lata J Gul-Hinc S Bielarczyk H Ronowska A Zysk M Gruzewska K. Differential effects of lipopolysaccharide on energy metabolism in murine microglial N9 and cholinergic SN56 neuronal cells. J Neurochem. (2015) 133:284–97. 10.1111/jnc.12979 25345568

[146] Orihuela R McPherson CA Harry GJ. Microglial M1/M2 polarization and metabolic states. Br J Pharmacol. (2016) 173:649–65. 10.1111/bph.13139 25800044 PMC4742299

[147] Van den Bossche J Baardman J de Winther MP Metabolic characterization of polarized M1 and M2 bone marrow-derived macrophages using real-time extracellular flux analysis. J Visual Exp. (2015) 105:e53424 10.3791/53424 PMC4692751 26649578

[148] Vats D Mukundan L Odegaard JI Zhang L Smith KL Morel CR. Oxidative metabolism and PGC-1beta attenuate macrophage-mediated inflammation. Cell Metab. (2006) 4:13–24. 10.1016/j.cmet.2006.05.011 16814729 PMC1904486

[149] Cherry JD Olschowka JA O'Banion MK. Neuroinflammation and M2 microglia: the good, the bad, and the inflamed. J Neuroinflam. (2014) 11:98. 10.1186/1742-2094-11-98 24889886 PMC4060849

[150] Tyrrell DJ Bharadwaj MS Jorgensen MJ Register TC Shively C Andrews RN. Blood-based bioenergetic profiling reflects differences in brain bioenergetics and metabolism. Oxid Med Cell Longevity (2017) 2017:7317251. 10.1155/2017/7317251 29098063 PMC5643153

[151] Prabakaran S Swatton JE Ryan MM Huffaker SJ Huang JT Griffin JL. Mitochondrial dysfunction in schizophrenia: evidence for compromised brain metabolism and oxidative stress. Mol Psychiatry (2004) 9:684–97, 643. 10.1038/sj.mp.4001511 15098003

[152] Karabatsiakis A Bock C Salinas-Manrique J Kolassa S Calzia E Dietrich DE. Mitochondrial respiration in peripheral blood mononuclear cells correlates with depressive subsymptoms and severity of major depression. Transl Psychiatry (2014) 4:e397. 10.1038/tp.2014.44 26126180 PMC4080325

[153] Hroudova J Fisar Z Kitzlerova E Zverova M Raboch J. Mitochondrial respiration in blood platelets of depressive patients. Mitochondrion (2013) 13:795–800. 10.1016/j.mito.2013.05.005 23688905

[154] Gubert C Stertz L Pfaffenseller B Panizzutti BS Rezin GT Massuda R. Mitochondrial activity and oxidative stress markers in peripheral blood mononuclear cells of patients with bipolar disorder, schizophrenia, and healthy subjects. J Psychiatr Res. (2013) 47:1396–402. 10.1016/j.jpsychires.2013.06.018 23870796

[155] Lopez-Armada MJ Riveiro-Naveira RR Vaamonde-Garcia C Valcarcel-Ares MN. Mitochondrial dysfunction and the inflammatory response. Mitochondrion (2013) 13:106–18. 10.1016/j.mito.2013.01.003 23333405

[156] Ng DS Chu T Esposito B Hui P Connelly PW Gross PL. Paraoxonase-1 deficiency in mice predisposes to vascular inflammation, oxidative stress, and thrombogenicity in the absence of hyperlipidemia. Cardiovasc Pathol. (2008) 17:226–32. 10.1016/j.carpath.2007.10.001 18402813

[157] Chacko BK Kramer PA Ravi S Benavides GA Mitchell T Dranka BP. The Bioenergetic Health Index: a new concept in mitochondrial translational research. Clin Sci. (2014) 127:367–73. 10.1042/CS20140101 24895057 PMC4202728

[158] Chacko BK Zhi D Darley-Usmar VM Mitchell T. The Bioenergetic Health Index is a sensitive measure of oxidative stress in human monocytes. Redox Biol. (2016) 8:43–50. 10.1016/j.redox.2015.12.008 26748041 PMC4712317

